# Effectiveness and Safety of Manufactured Chinese Herbal Formula for Knee Osteoarthritis: Insights from a Systematic Review

**DOI:** 10.1155/2015/328642

**Published:** 2015-11-01

**Authors:** Liguo Zhu, Shaofeng Yang, Shangquan Wang, Hao Gong, Linghui Li, Xu Wei

**Affiliations:** ^1^Department of Spine, Wangjing Hospital, China Academy of Chinese Medical Sciences, Huajiadi Street, Chaoyang District, Beijing 100102, China; ^2^Department of Spine, The First Hospital of Hunan University of Chinese Medicine, No. 95 Middle Shaoshan Street, Yuhua District, Changsha 410007, China; ^3^Department of General Orthopedics, Wangjing Hospital, China Academy of Chinese Medical Sciences, Huajiadi Street, Chaoyang District, Beijing 100102, China; ^4^Department of Orthopaedics, Changping Hospital of Integrated Chinese and Western Medicine, No. 219 Huangping Street, Changping District, Beijing 102208, China; ^5^Department of Scientific Research, Wangjing Hospital, China Academy of Chinese Medical Sciences, Huajiadi Street, Chaoyang District, Beijing 100102, China

## Abstract

*Objective*. To assess the current clinical evidence of manufactured Chinese herbal formulae (MCHF) for knee osteoarthritis (KOA). * Methods*. Seven databases were searched from inception to May 2015. Eligible randomized controlled trials investigating the effectiveness of MCHF for KOA were included. Data extraction, methodological assessment, and meta-analyses were conducted according to the Cochrane standards. *Results*. A total of 17 kinds of MCHF were identified from the twenty-six included trials. Meta-analyses showed that MCHF significantly relieved the global pain of knee joints, either used alone or combined with routine treatments. Additionally, MCHF plus routine treatments significantly decreased the scores of WOMAC and Lequesne index. However, there were no statistical differences between MCHF group and routine treatment group in walk-related pain and WOMAC scores. No significant differences were found in Lysholm scores. There were twenty-one trials that mentioned adverse events. A pooled analysis showed that adverse events occurred more frequently in control group compared with MCHF group. *Conclusions*. Our results indicated that MCHF showed some potential benefits for KOA. However, we still cannot draw firm conclusions due to the poor methodological quality of included trials. More high-quality RCTs would help to confirm the evidence.

## 1. Introduction

Knee osteoarthritis (KOA) is a serious rheumatic disease in which the cartilage breaks down and causes the narrowing of joint space. In severe conditions, when knee joints completely lose cartilage, the periarticular bone and soft tissue structures would start to change and this may cause joint pain, swelling, misshapenness, and disability [1, 2]. In America, symptomatic KOA occurs in approximately 10% of adults aged 60 years or older [3]. According to recent statistics, there were 9.3 million adults who suffered from KOA in the United States [4]. As a consequence of the aging process, the number of people with KOA is expected to increase in the next decades [5, 6]. Osteoarthritis is predicted to be the fourth leading cause of disability by 2020 [7].

The aims of management for KOA are to relieve pain or swelling, improve mobility of the joint, and minimize disability. The current treatment options recommended in several evidence-based clinical guidelines include weight loss, exercises, oral pharmacological medications, topical therapies, intra-articular therapies, nonpharmacological therapies, and surgical treatments [8–10]. Among these various interventions, nonsteroidal anti-inflammatory drugs and intra-articular hyaluronic acid or corticosteroids were more commonly used in clinical practice [11, 12]. However, such treatments may be ineffective or lead to serious adverse events in some patients [13]. Under this circumstance, clinicians and patients were becoming more willing to adopt complementary and alternative medicine as a treatment option for KOA [14–16].

In mainland China and increasingly worldwide, Chinese herbal medicine has been used in varied forms for the treatment of KOA [17–19]. Manufactured Chinese herbal formulae (MCHF), also known as Chinese patent medicine, combined several Chinese herbs together to improve the therapeutic effects and reduce the side effects [20]. Hundreds of MCHF were approved by China Food and Drug Administration (CFDA) and listed in the “National Essential Drug List” (2012 Edition), providing basic medical support for patients in China. Although the mechanism has not been fully illuminated, animal experimental studies indicated that Chinese herbs decreased the levels of nitric oxide in the serum, synovium, and joint cartilage in OA rabbits [21]. Another research showed that Du-huo-ji-sheng decoction (a traditional Chinese herbal medicine) significantly exerted therapeutic effects on OA rabbits, probably through inhibiting the expressions of vascular endothelial growth factor and hypoxia inducible factor-1*α* [22].

Scientificity is a big obstacle for the acceptance of Chinese medicine in western countries. Previous meta-analyses have been conducted to evaluate oral or topical applications of herbal medicine in treating osteoarthritis [23, 24]. So far, there has been no systematic review of randomized controlled trials of MCHF in treating KOA. Therefore, we performed a systematic review in an attempt to better define the efficacy and safety of MCHF in the treatment of KOA.

## 2. Methods

### 2.1. Eligibility Criteria

We included all published and unpublished randomized controlled trials (RCTs) testing the effectiveness of MCHF in the treatment of KOA. Studies should involve human subjects and original data should be presented. The patients should be diagnosed by standard criteria of KOA, for instance, American College of Rheumatology (ACR) criteria in 1995 [25], Chinese Medical Association criteria in 2007 [26], or European League against Rheumatism (EULAR) criteria in 2010 [27].

The interventions in experimental group were MCHF, either used alone or combined with routine treatments, regardless of dose and duration. Manufactured Chinese herbal formulae included in the trials should be approved by China Food and Drug Administration (CFDA). The control group received no treatment, placebo, or routine treatments. Routine treatments were in accordance with the recommendations in clinical practice guideline, such as the Osteoarthritis Research Society International (OARSI) guidelines [28]. Cotherapies including herbal medicine, acupuncture, moxibustion, manual therapy, tai chi, and yoga were excluded.

One or more of primary or secondary outcomes should be reported in the included RCTs. The primary outcomes were the pain and function of knee joint measured by recognized scales, including Visual Analog Score (VAS), Western Ontario and MacMaster universities arthritis index (WOMAC), Lequesne functional index (Lequesne), Lysholm knee score scale (Lysholm), or analogous pain scales. In this review, we focused on the total score of these scales. The secondary outcomes were adverse events and quality of life (36-item short form health survey, SF-36).

### 2.2. Database and Search Strategies

This review was guided by the PRISMA statement [55]. Studies were identified by electronic database search from inception until May 31, 2015, in PubMed, Cochrane Central Register of Controlled Trials (CENTRAL), Web of Science (SCI), https://clinicaltrials.gov/, China National Knowledge Infrastructure (CNKI), Wanfang Database (Wanfang), and Chinese Biomedical Literature Database (CBM). There were no restrictions on language or publication type. Titles and abstracts were scanned by two authors (Linghui Li and Hao Gong) independently to make an initial assessment based on the inclusion and exclusion criteria. Full-texts were assessed by two independent reviewers (Linghui Li and Hao Gong) when the information of titles and abstracts was not enough to judge the relevance. The reference list of retrieved papers was also searched. Disagreements were resolved by a third reviewer (Liguo Zhu). The strategy for searching PubMed was listed as follows:

(“osteoarthritis, knee” [MeSH Terms] OR (“osteoarthritis” [All Fields] AND “knee” [All Fields]) OR “knee osteoarthritis” [All Fields] OR (“knee” [All Fields] AND “osteoarthritis” [All Fields])) AND “Chinese” [All Fields].

### 2.3. Data Extraction and Quality Assessment

The following data were extracted: the first author names, year of publication, sample size, the characteristics of the patients (age and sex), diagnosis criteria, details of interventions in experimental and control groups, adverse events, and outcome measures for each study. Intention-to-treat data was extracted from the primary articles [56, 57]. Where authors indicated a trial protocol registry number, the protocol of previous study was retrieved. Supplementary table provided the full names and components of MCHF. Data extraction was conducted by two authors (Shaofeng Yang and Shangquan Wang) independently according to predefined criteria.

### 2.4. Quality Assessment

We adopted Cochrane Collaboration's tool for assessing risk of bias [58]. Six different domains, including a random sequence generation (selection bias), allocation concealment (selection bias), blinding of participants and personnel (performance bias), blinding of outcome assessment (detection bias), incomplete outcome data (attrition bias), and selective outcome reporting (reporting bias), and other potential sources of bias were used to evaluate methodological quality of included studies. Each study was judged to be low, high, or unclear risk of bias. Two reviewers (Linghui Li and Xu Wei) independently assessed the quality of the included studies. A third reviewer (Liguo Zhu) resolved discrepancies.

### 2.5. Data Synthesis

We performed a meta-analysis using RevMan (version 5.3) [59]. For continuous data of our review, the difference in change from baseline to posttreatment between two groups was expressed as mean differences (MD) or standardized mean differences (SMD) with 95% confidence interval (CI). For dichotomous data, the effect of the intervention was expressed as relative risk (RR) with 95% CI. Random or fixed-effect model was used to calculate summary effects estimates [60] and heterogeneity was assessed using the I2 statistic which showed the proportion of variability between effect estimates [61]. One reason for high heterogeneity may be due to variability in the routine treatments. So we considered subgroup analysis to explore heterogeneity. Forest plots were created to depict estimates and confidence intervals of the interventions for each study. And plotting symbol size represented the weight of included studies in the meta-analysis [62]. Asymmetry and publication bias of the studies were evaluated by a funnel plot [63].

## 3. Results

### 3.1. Description of Included Trials

**Figure 1:**
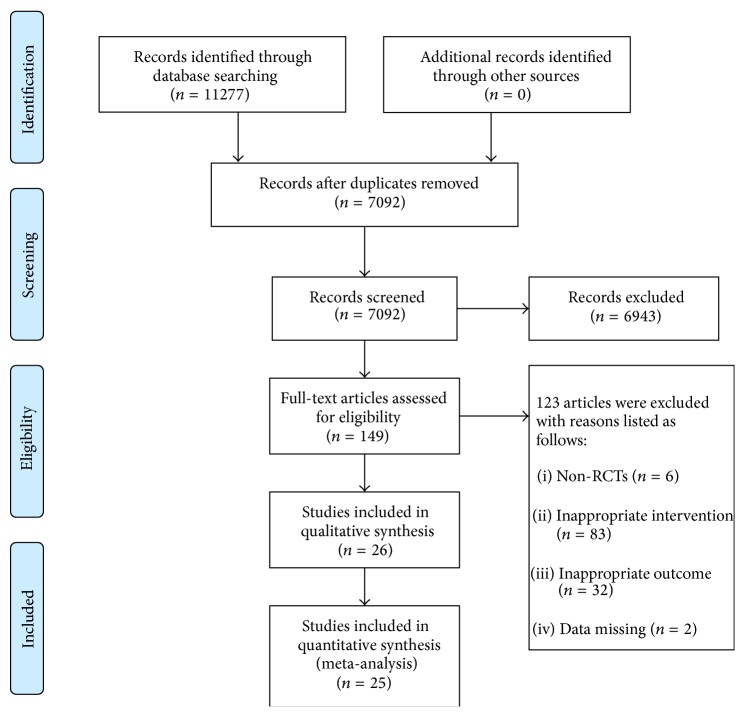
Flow chart of study search and selection.

A total of 11277 records were identified through electronic databases: CNKI (*n* = 4356), CBM (*n* = 4565), Wanfang data (*n* = 1910), Medline (*n* = 255), CENTRAL (*n* = 113), SCI (*n* = 63), and https://clinicaltrials.gov/ (*n* = 15). After removing duplicates, we screened 7092 records through titles and abstracts and selected 149 for full-text assessment. There were 6 nonrandomized controlled trials, 115 trials with inappropriate interventions or outcomes, and 2 trials with incomplete data. In the end, twenty-six trials (all conducted in China and published in Chinese) met the inclusion criteria (Figure 1). Searches for trial registries yielded no ongoing trials. The characteristics of the 26 included studies are given in Table 1.

Twenty-six randomized controlled studies with 32 comparisons involving 3472 participants (1846 in experimental group and 1626 in control group) with KOA met the criteria for the current review. The diagnoses of KOA were according to the American College of Rheumatology criteria, the Chinese Medical Association criteria, or Guidelines for Clinical Research of New Drugs of Traditional Chinese Medicine (GCRNDTCM). Specific diagnostic criteria in two trials were not reported [35, 48]. The patients ranged from 38 to 79 years old. Baseline demographic characteristics (age, sex, and disease course) were provided in all included studies except one [34] and all showed no significant differences between groups.

All trials used MCHF alone or combined with routine treatments in the experimental group. Seventeen kinds of MCHF were involved in the current review (as shown in Table 2), six of which were recorded in China Pharmacopoeia (2015 Edition) with the indication of chronic joint pain rather than the standard name of KOA. All of the included MCHF were approved by China Food and Drug Administration (CFDA) and were commercially available. The interventions for control group were only routine treatments, including oral nonsteroidal anti-inflammatory drugs (NSAIDs: diclofenac sodium, aceclofenac, diacerein, ibuprofen, indometacin, ketoprofen, celecoxib, naproxen, and nabumetone), oral glucosamine, intra-articular injection (sodium hyaluronate and ozone), and nonpharmacotherapy (infrared therapy). The treatment duration varied from 15 days to 3 months.

Participants in four trials [31, 39, 40, 46] were divided into three groups comparing MCHF used alone and combined with routine treatments to routine treatments. One trial [38] had four intervention arms (MCHF versus MCHF plus routine treatments versus diclofenac sodium versus ozone). One trial [47] had three intervention arms (MCHF versus glucosamine versus Shi's manipulation), so only the first two related arms comparing MCHF to routine treatments were included. The other twenty-one trials were all randomized controlled trials with two groups comparing MCHF intervention alone or MCHF plus routine treatments to routine treatments alone. Various outcomes were compared between different treatment groups (as shown in Table 1).

### 3.2. Methodological Quality

The methodological quality in the included studies was assessed according to the criteria in the* Cochrane Handbook for Systematic Review of interventions*. The methodological quality of the majority studies was “poor.” All of the included studies stated randomization; however only 13 trials mentioned the specific methods for sequence generation, such as drawing of lots [31], computer software [39], stratified randomization [46], and random number table. Allocation concealment was only mentioned in one trial [29]. One trial was of double-blind design [30] and one trial was not blinded [29]. The rest of trials did not report any information on blinding at all. Only eight trials reported information on drop-out. Three trials conducted follow-ups of 8 weeks [31, 39] and 1 year [51]. Selective reporting was unclear due to the unavailability of the research proposal. None of the studies described sample size calculation. Risk of bias summary of included studies was shown in Table 3.

### 3.3. Effect on Joint Pain

There were sixteen trials reporting pain VAS as primary outcome measure. However the reporting of measures was different in these studies, which could limit the utility of studies for meta-analysis. So we conducted meta-analyses as two parts: VAS-1 (global pain) and VAS-2 (pain on walking). VAS pain scale was generally considered as 10 cm lines from 0 (nil symptom) to 10 (worst possible symptom), but in several trials it is reported on a centimeter scale in the range from 0 to 100. We converted these outcomes to the 0 to 10 cm scale in meta-analyses. One reason for high heterogeneity may be due to variability in the routine treatments, so subgroup analyses were conducted to explore the heterogeneity.

#### 3.3.1. Visual Analog Score-1 (Global Pain)

**Figure 2:**
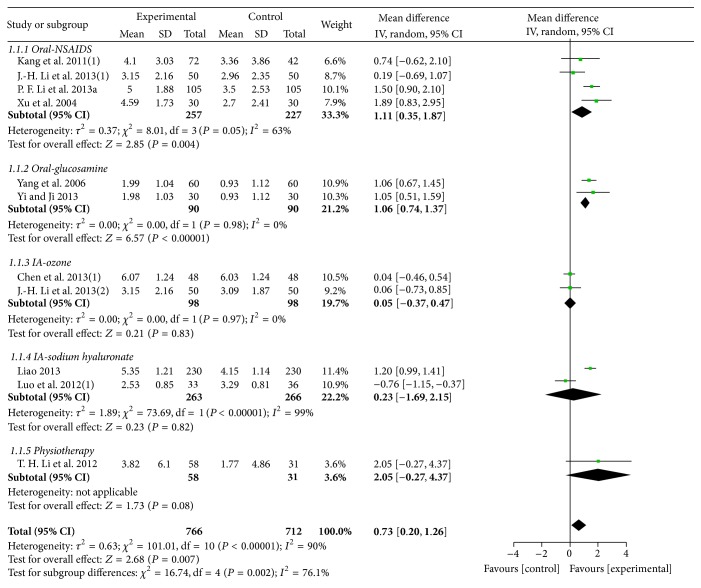
Forest plot of comparison: MCHF versus routine treatment for KOA, outcome: VAS-1 (global pain).

Ten trials with 11 comparisons (involving 1478 participants) reported the effects of MCHF alone versus routine treatments on pain VAS-1. These trials have statistical heterogeneity in the consistency of the results, so we used a random effects model for meta-analysis (*χ* ^2^ = 101.01, *P* < 0.00001; *I* ^2^ = 90%). The results showed significant beneficial effects of MCHF used alone compared with routine treatment (MD: 0.73 [0.20, 1.26]; *P* = 0.007). Subgroup analysis revealed that MCHF could reduce global pain of knee joint when compared with oral nonsteroidal anti-inflammatory drugs (NSAIDs) (MD: 1.11 [0.35, 1.87]; *P* = 0.004) and oral glucosamine (MD: 1.06 [0.74, 1.37]; *P* < 0.00001), but no statistically significant differences were observed when compared with intra-articular injection therapy (ozone: MD: 0.05 [−0.37, 0.47], *P* = 0.83; sodium hyaluronate: MD: 0.23 [−1.69, 2.15]; *P* = 0.82) or physiotherapy (MD: 2.05 [−0.27, 4.37]; *P* = 0.08) (as shown in Figure 2).

**Figure 3:**
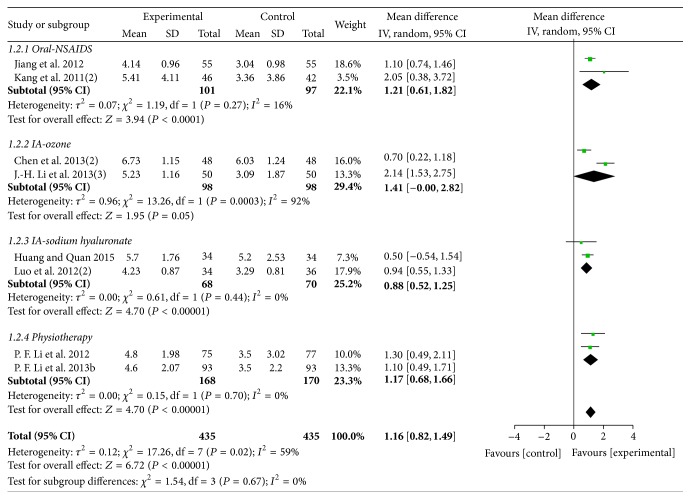
Forest plot of comparison: MCHF plus routine treatment versus routine treatment for KOA, outcome: VAS-1 (global pain).

Eight trials (involving 870 participants) compared the effects of MCHF plus routine treatments to routine treatments. A random effects model was used for meta-analysis due to the significant heterogeneity (*χ* ^2^ = 17.26, *P* = 0.02; *I* ^2^ = 59%). The results showed that MCHF plus routine treatments significantly reduced the mean VAS-1 score compared with routine treatments (MD: 1.16 [0.82, 1.49]; *P* < 0.00001), such as NSAIDs (MD: 1.21 [0.61, 1.82]; *P* < 0.0001), physiotherapy (MD: 1.17 [0.68, 1.66]; *P* < 0.00001), and intra-articular injection of sodium hyaluronate (MD: 0.88 [0.52, 1.25]; *P* < 0.00001) or ozone (MD: 1.41 [−0.00, 2.82]; *P* = 0.05) (as shown in Figure 3).

#### 3.3.2. Visual Analog Score-2 (Pain on Walking)

**Figure 4:**
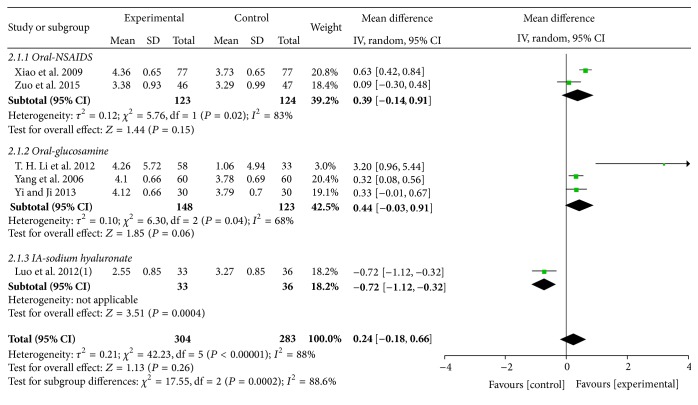
Forest plot of comparison: MCHF individually versus routine treatment for KOA, outcome: VAS-2 (pain on walking).

A total of six trials with 7 comparisons (involving 657 participants) reported pain VAS-2 as outcome measure. Among the six trials, one trial [40] compared the effects of both MCHF used alone and combined with routine treatments to routine treatments (sodium hyaluronate injection). The results showed no significant differences between MCHF group and sodium hyaluronate injection group. However, MCHF plus sodium hyaluronate injection treatments significantly reduced the average pain score compared with both MCHF and sodium hyaluronate injection used alone. The other five trials [29, 33, 42, 48, 53] all compared the effects of MCHF used alone to routine treatments. A random effects model was used for meta-analysis (*χ* ^2^ = 42.23, *P* < 0.00001; *I* ^2^ = 88%) and the two groups showed similar efficacy in reducing VAS-2 scores (MD: 0.24 [−0.18, 0.66]; *P* = 0.26). Subgroup analysis showed no statistically significant differences between MCHF group and oral drug group (NSAIDs: MD: 0.39 [−0.14, 0.91], *P* = 0.15; glucosamine: MD: 0.44 [−0.03, 0.91]; *P* = 0.06). Compared to MCHF group, a better therapeutic effect in relieving walk-associated pain was found in sodium hyaluronate injection group (MD: −0.72 [−1.12, −0.32]; *P* = 0.0004) (as shown in Figure 4).

### 3.4. Effect on Joint Function

Physical function of knee joints was measured by WOMAC, Lequesne, Lysholm scores, and Hospital for Special Surgery (HSS) scales. Subgroup analyses were conducted to explore heterogeneity (based on various routine treatments).

#### 3.4.1. WOMAC

WOMAC was used as outcome measure in six trials [29, 32, 38, 40, 41, 47]. However, there were two scoring methods used for the scale. These two scoring methods were both acceptable for clinical researches, so mean difference (MD) was replaced by standardized mean difference (SMD) for meta-analyses.

**Figure 5:**
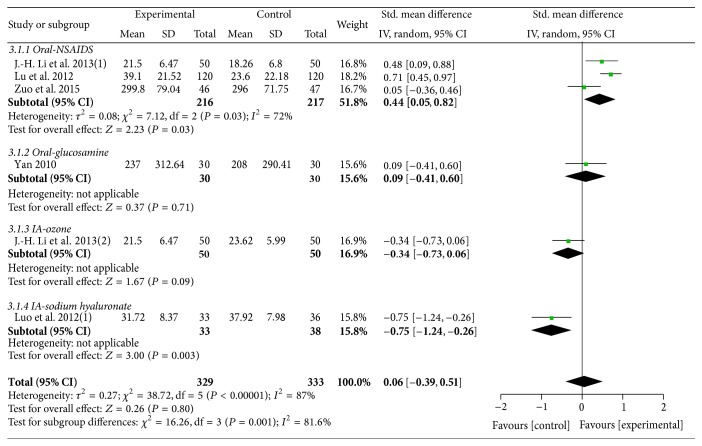
Forest plot of comparison: MCHF individually versus routine treatment for KOA, outcome: WOMAC score.

Five trials [29, 38, 40, 41, 47] with 6 comparisons including 662 participants reported the effect of MCHF individually versus routine treatments. A random effects model was used for meta-analysis (*χ* ^2^ = 38.72, *P* < 0.00001; *I* ^2^ = 87%). No significant differences were found between the two groups on WOMAC scores (SMD: 0.06 [−0.39, 0.51]; *P* = 0.80). Subgroup analysis revealed a better therapeutic effect in experimental group compared with oral NSAIDs group (SMD: 0.44 [0.05, 0.82]; *P* = 0.03). No statistically significant differences were found when compared with oral glucosamine (SMD: 0.09 [−0.41, 0.60]; *P* = 0.71) or ozone injection (SMD: −0.34 [−0.73, 0.06]; *P* = 0.09). The therapeutic effect of MCHF fell short of sodium hyaluronate injection in WOMAC scores (SMD: −0.75 [−1.24, −0.26]; *P* = 0.003) (as shown in Figure 5).

**Figure 6:**
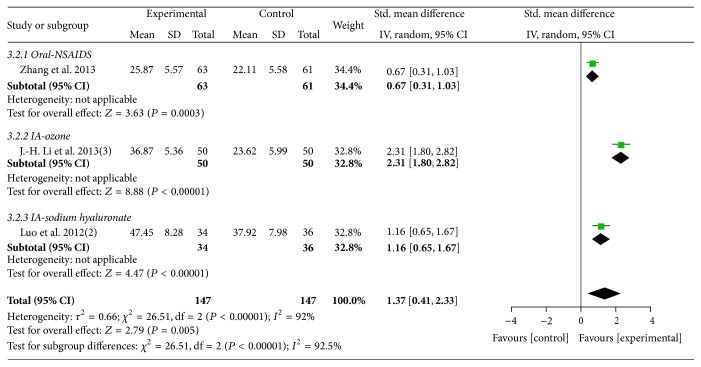
Forest plot of comparison: MCHF plus routine treatments versus routine treatment for KOA, outcome: WOMAC score.

Three trials [32, 38, 40] (involving 294 participants) contributed to the meta-analysis of comparison of MCHF plus routine treatments versus routine treatments (as shown in Figure 6). The results showed favorable effect of MCHF plus routine treatments compared to routine treatments (total: SMD: 1.37 [0.41, 2.33]; *P* = 0.005; oral NSAIDs group: SMD: 0.67 [0.31, 1.03]; *P* = 0.0003; ozone injection group: SMD: 2.31 [1.80, 2.82]; sodium hyaluronate injection group: SMD: 1.16 [0.65, 1.67]).

#### 3.4.2. Lequesne

Three trials [31, 34, 45] used Lequesne algofunctional index as outcome measure. One trial [31] compared the effects of MCHF both used alone and combined with routine treatments to routine treatments (oral NSAIDs therapy). The results showed no significant differences between MCHF group and oral NSAIDs therapy group.

**Figure 7:**
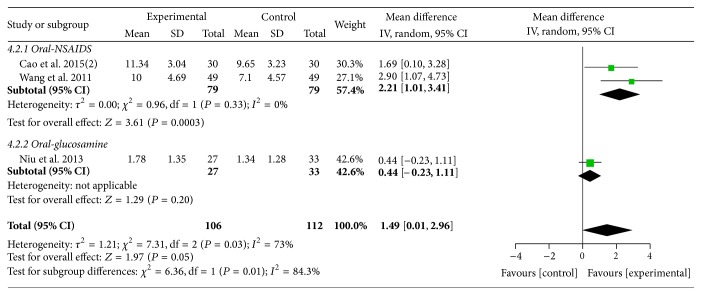
Forest plot of comparison: MCHF plus routine treatments versus routine treatment for KOA, outcome: Lequesne index.

Three trials [31, 34, 45] (involving 218 participants) contributed to the meta-analysis of comparison of MCHF plus routine treatments versus routine treatments. A random effects model was used for meta-analysis (*χ* ^2^ = 7.31, *P* = 0.03; *I* ^2^ = 73%). The results showed small beneficial effects of MCHF plus routine treatments compared to routine treatments (MD: 1.49 [0.01, 2.96]; *P* = 0.05) (as shown in Figure 7). Subgroup analysis revealed a better therapeutic effect in experimental group compared with oral NSAIDs group (MD: 2.21 [1.01, 3.41]; *P* = 0.0003), but no significantly differences were found when compared with oral glucosamine group (MD: 0.44 [−0.23, 1.11]; *P* = 0.20).

#### 3.4.3. Lysholm

**Figure 8:**
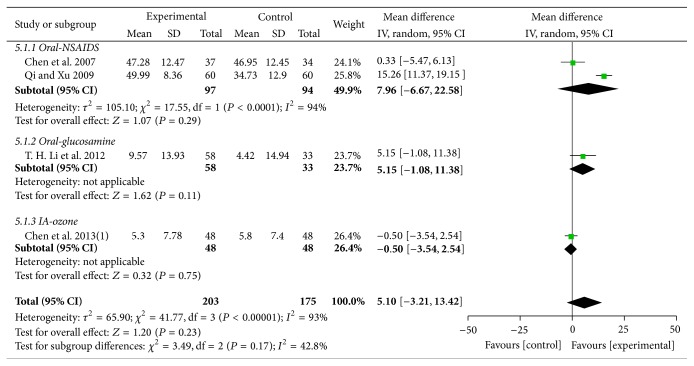
Forest plot of comparison: MCHF individually versus routine treatment for KOA, outcome: Lysholm score.

**Figure 9:**
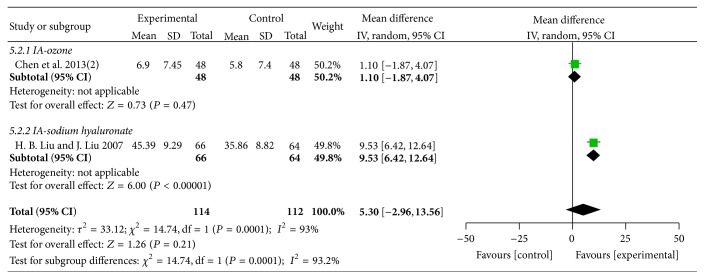
Forest plot of comparison: MCHF plus routine treatments versus routine treatment for KOA, outcome: Lysholm score.

Five trials [39, 42, 49, 51, 52] with six comparisons involving 604 participants used Lysholm as outcome measure. One trial [39] compared the effects of MCHF both used alone and combined with routine treatments to routine treatments. As shown in the result of meta-analyses, no significant differences were found in the comparisons of MCHF individually versus routine treatments (MD: 5.10 [−3.21, 13.42]; *P* = 0.23) (as shown in Figure 8) and MCHF plus routine treatments versus routine treatments (MD: 5.30 [−2.96, 13.56]; *P* = 0.21) (as shown in Figure 9). Subgroup analysis revealed significant improvements on Lysholm scores in MCHF plus sodium hyaluronate injection group, compared with sodium hyaluronate injection used alone (MD: 9.53 [6.42, 12.64]; *P* < 0.00001), but no significantly differences were found in other comparisons.

#### 3.4.4. HSS

One trial [50] involving 100 participants compared 6 weeks of intervention with MCHF (Bi qi capsule) against glucosamine (250 mg, tid). As reported, participants in both groups showed significant improvements in HSS score. However, between-group differences were not statistically significant suggesting that Bi qi capsule has comparable efficacy to glucosamine in the improvement of physical function of knee joints.

### 3.5. Effect on Quality of Life

Two trials [29, 34] involving 153 participants measured quality of life by SF-36. They assessed the general health concepts by the following eight subscales: physical functioning, role limitations due to physical health problems, bodily pain, general health perceptions, vitality, social functioning, role limitations due to emotional problems, and mental health. Data from one trial [29] showed no statistically significant differences in each subscale between MCHF group and routine treatment group (*P* > 0.05). However, the result for the comparison of MCHF plus routine treatments versus routine treatments revealed that the SF-36 scores were significantly increased in experimental group (*P* < 0.05).

### 3.6. Adverse Events

Twenty-one trials (involving 2836 patients) mentioned the occurrence or absence of adverse events and the other four trials [36, 43, 50, 53] provided no data about adverse events. Six trials [34, 37, 42, 45, 51, 52] reported that there were no adverse events in both experimental group and control group. The reported adverse events included gastrointestinal symptoms, abnormal liver function, dry mouth, local swelling, dizziness, and eruption. The majority of adverse events were not severe and they spontaneously recovered after drug discontinuance or symptomatic treatment. No serious adverse event was reported.

**Figure 10:**
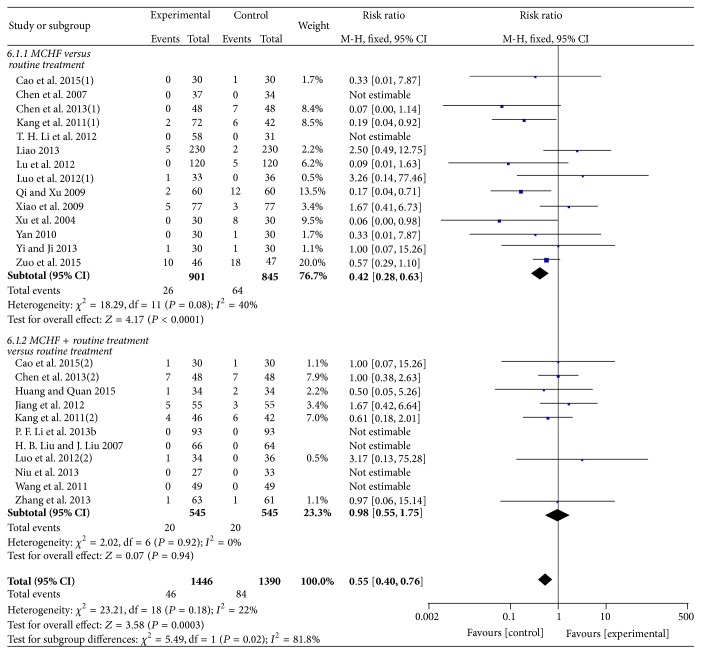
Forest plot of comparison: MCHF alone or MCHF plus routine treatments versus routine treatment for KOA, outcome: adverse events.

Meta-analysis was conducted to compare the adverse events in experimental group and control group. Fixed-effects models were used for meta-analysis (MCHF versus routine treatments: *χ* ^2^ = 18.29, *P* = 0.08; *I* ^2^ = 40%; MCHF plus routine treatment versus routine treatment: *χ* ^2^ = 2.02, *P* = 0.92; *I* ^2^ = 0%). No significant differences were found in adverse events between MCHF plus routine treatments group and routine treatments group (RR = 0.98 [0.55, 1.75]; *P* = 0.94). However the result for the comparison of MCHF alone versus routine treatments revealed that adverse events occurred more frequently in control group (RR = 0.42 [0.28, 0.63]; *P* < 0.0001) (as shown in Figure 10).

### 3.7. Publication Bias

**Figure 11:**
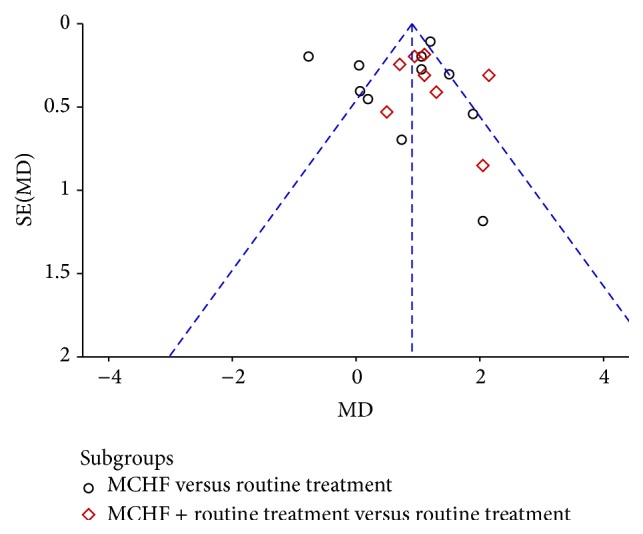
Funnel plot of comparison: MCHF alone or MCHF plus routine treatments versus routine treatment for KOA, outcome: VAS-1 (global pain).

A funnel plot revealed an asymmetrical distribution of studies comparing MCHF (used alone or combined with routine treatments) to routine treatments for global pain of KOA (Figure 11). These nineteen trials were all published in Chinese and most of them reported positive results, which indicated that there is publication bias.

## 4. Discussions

### 4.1. Summary of Evidence

Manufactured Chinese herbal formulae (MCHF), also known as Chinese patent medicine, are typical combination of several Chinese herbals which could enhance the therapeutic effect and reduce the side effect of a single component. The quality control and approval for marketing of MCHF are conducted by CFDA. It is commercially available and widely used in mainland China due to the convenience of application [64–66]. In recent years, more and more researchers at home and abroad have paid great attention to MCHF for their remarkable effect and enormous market share [67–69]. It is reported that some MCHF (Yaotongning capsule and Tougu Xiaotong capsule) promoted the proliferation and glycosaminoglycan synthesis in IL-1*β* induced chondrocytes and may have the potential activity on treating chondrocytes degeneration caused by osteoarthritis [70, 71]. A large number of clinical trials reported that MCHF could alleviate the symptoms of KOA [72]. It is important to perform objective reviews to assess the clinical evidence of MCHF for KOA.

Several reviews provided evidence for the effectiveness of oral and topical use of herbal medicines for osteoarthritis. For example, one study investigated the effect of traditional Chinese herbal patches in the treatment of osteoarthritis [24]. There were 86 kinds of TCHPs identified in the review and the result showed certain evidence of TCHPs in improving the global effectiveness rate for OA. One study [23] assessed the effect of oral medicinal plant products used in various countries for osteoarthritis. There were 31 single medicinal plant products and several polyherbal preparations identified in 49 studies. However, meta-analyses were only possible for two kinds of herbal products due to the different study protocols and interventions. All other herbal medicines in this review were investigated in single trials, limiting conclusions. To date, the role of MCHF for KOA is still unknown. Therefore, the current review aims to investigate both of the therapeutic benefits and the safety concerns of MCHF.

The current systematic review included 26 randomized controlled trials involving 3472 participants with KOA. All trials used MCHF alone or combined with routine treatments in the experimental group. The interventions for control group were routine treatments, such as oral drugs (NSAIDs and glucosamine), intra-articular injection (sodium hyaluronate and ozone), and nonpharmacotherapy (infrared therapy). A total of 17 kinds of MCHF were identified and six of them were recorded in China Pharmacopoeia (2015 Edition) with the indication of chronic joint pain rather than the standard name of KOA. It is reported in previous studies that the clinical efficacy of MCHF (Kang gu zeng sheng tablets) in KOA might be related to regulation of basic Fibroblast Growth Factor protein and mRNA expression in chondrocytes [73]. One study [74] claimed that MCHF (Bi qi capsule) could protect articular cartilage of patients with KOA by reducing the activity of MMP-3 in serum and synovial fluid, improving the activity of TIMP-1 and inhibiting the degradation of cartilage matrix.

Meta-analyses revealed that MCHF significantly relieved global pain of knee joint when compared with routine treatments (medication alone: MD: 0.73 [0.20, 1.26]; *P* = 0.007; combination treatment: MD: 1.16 [0.82, 1.49]; *P* < 0.00001) but showed similar efficacy to control group in alleviating walk-related pain (MD: 0.24 [−0.18, 0.66]; *P* = 0.26). There were no beneficial effects in improving the physical function of knee joints when using MCHF alone. However, the combination of MCHF and routine treatments showed remarkable curative effects in improving knee joint functions (WOMAC: SMD: 1.37 [0.41, 2.33]; *P* = 0.005; Lequesne: MD: 1.49 [0.01, 2.96]; *P* = 0.05). There was no serious adverse event reported in the included trials. According to the existing data, adverse events occurred more frequently in routine treatment group (RR = 0.55 [0.40, 0.76]; *P* = 0.0003). Since four of twenty-six trials provided no data about adverse events, we still cannot draw definitive conclusions about the safety of MCHF.

### 4.2. Limitations

This study is the first systematic review of MCHF in treating KOA. We performed the review referring to the PRISMA through all stages to ensure the research quality. The results of meta-analysis showed that MCHF seemed to be an effective and safe intervention for global pain relief and function improvement of knee joint. However, there were several limitations existing in this review. Though we performed a thorough search strategy in seven electronic databases, we still cannot be absolutely certain that all relevant trials were found. All of the included trials were conducted in China and published in Chinese. Only KOA was referred to in our studies, so conclusions regarding osteoarthritis of other joints could not be drawn from our systematic review. Even though all the included trials mentioned randomization, half of them did not describe the specific methods of random sequence generation. Allocation concealment and blinding were only mentioned in one trial. The treatment duration in this review was short, varying from 15 days to 3 months. The majority of included trials did not report information on drop-out and follow-ups. Selective reporting was unclear in all trials due to the unavailability of the research proposal. None of the included trials reported a pretrial estimation of sample size. The biases of selection, detection, implementation, and publication should be taken into consideration when interpreting the conclusion in this review.

## 5. Conclusion

The current review indicates that MCHF have considerable therapeutic effect in the treatment of KOA with no serious side effects. Meta-analyses showed that MCHF seemed to be an effective method for global pain relief, either alone or in combination. In addition, MCHF combined with routine treatments appeared to be more effective in improving the physical function of knee joints compared with routine treatments. There was a slightly raised risk of adverse events in control group compared with MCHF group. However, we still cannot draw definitive conclusions due to the poor methodological quality of included studies. More high-quality RCTs would help clarify the definite efficacy and safety of MCHF in the treatment of KOA.

## Figures and Tables

**Table 1:** Characteristics of included studies on manufactured Chinese herbal formulae for KOA.

Study ID	Sample size (EG/CG)	Age (yrs) (mean)	Sex (male/female)	Diagnostic criteria	Intervention		Adverse events (cases)	Outcomes
					Experimental group	Control group		
Zuo et al. (2015) [29]	46/47	EG: 56.22 ± 9.92 CG: 58.58 ± 8.84	EG: 7/34 CG: 6/32	ACR	Fufang Xiatianwu pill (0.6 g, tid, 12 w)	Diclofenac sodium (25 mg, tid, 12 w)	EG: GS (10) CG: GS (18)	VAS-2; SF-36; WOMAC;

Huang and Quan (2015) [30]	34/34	EG: 53–76 (68) CG: 53–76 (65)	EG: 19/15 CG: 18/16	ACR	Huo xue zhi tong capsule (2 pills, tid, 4 w) + CG	Sodium hyaluronate (IA, 2 mL, qw, 4 w)	EG: GS (1) CG: GS (2)	VAS-1

Cao et al. (2015) [31]	EG1/EG2/CG: 30/30/30	EG1: 61.57 ± 6.68 EG2: 61.03 ± 5.89 CG: 61.41 ± 7.15	EG1: 8/22 EG2: 9/21 CG: 6/24	CMA (2007)	EG1: Jin tian ge capsule (3 pills, tid, 1 m); EG2: EG1 + CG	Aceclofenac (0.1 g, bid, 1 m);	EG1: None EG2: GS (1) CG: GS (1)	Lequesne

Zhang et al. (2013) [32]	63/61	EG: 38–70 (56.3) CG: 39–73 (57.6)	EG: 13/50 CG: 12/49	ACR (1995)	Pan long qi pill (0.9 g, tid, 4 w) + CG	Diacerein (50 mg, bid, 4 w) + Aceclofenac (0.1 g, bid, 4 w)	EG: GS (1); CG: ALF (1)	WOMAC

Yi and Ji (2013) [33]	30/30	EG: 40–70 (54.5) CG: 39–72 (55.3)	EG: 9/21 CG: 8/24	ACR	Pan long qi pill (3 pills, tid, 6 w)	Glucosamine (250 mg, tid, 6 w)	EG: GS (1) CG: GS (1)	VAS-1; VAS-2

Niu et al. (2013) [34]	27/33	NR	NR	CMA (2003)	Jin gu tong xiao pill (6 g, bid, 1 m) + CG	Glucosamine (2 pills, tid, 1 m)	None	Lequesne; SF-36

Liao (2013) [35]	230/230	EG: 57.67 ± 7.62 CG: 57.43 ± 7.81	EG: 105/125 CG: 101/129	NR	Jiangu granule (12 g, tid, 1 m)	Sodium hyaluronate (IA, 20 mg, qw, 1 m)	EG: DM (3); GS (2) CG: DM (1); GS (1)	VAS-1

Li et al. (2013) [36]	105/105	EG: 38–54 (45.0) CG: 40–52 (46.3)	EG: 70/35 CG: 72/33	ACR (1995)	Hua mo yan granule (12 g, tid, 4 w)	Ketoprofen (50 mg, tid, 4 w)	NR	VAS-1

Li et al. (2013) [37]	93/93	EG: 56.2 ± 6.7 CG: 54.8 ± 7.2	EG: 40/53 CG: 38/55	CMA (2007)	Hua mo yan granule (12 g, tid, 4 w) + CG	Infrared therapy (15 min, bid)	None	VAS-1

Li et al. (2013) [38]	EG1/EG2/CG1/CG2: 50/50/50/50	EG1: 64.23 ± 1.69 EG2: 64.56 ± 1.75 CG1: 64.35 ± 1.78 CG2: 63.85 ± 1.72	EG1: 22/28 EG2: 23/27 CG1: 23/27 CG2: 24/26	ACR	EG1: Fu gui gu tong granule (5 g, tid, 1 m) EG2: EG1 + CG2	CG1: Diclofenac sodium (25 mg, tid, 1 m) CG2: Ozone (IA, 10 mL, qw, 1 m)	GS	VAS-1; WOMAC

Chen et al. (2013) [39]	EG1/EG2/CG: 48/48/48	EG1: 58 ± 17.7 EG2: 61 ± 18.6 CG: 61 ± 16.0	EG1: 17/25 EG2: 15/25 CG: 14/28	CMA (2003)	EG1: Xian ling gu bao capsule (1.5 g, bid, 4 w) EG2: EG1 + CG	Ozone (IA, 15 mL, qw, 4 w)	EG1: None EG2: LS (7) CG: LS (7)	VAS-1; Lysholm

Luo et al. (2012) [40]	EG1/EG2/CG: 33/34/36	EG1: 41–74 (51.93) EG2: 42–67 (50.15) CG: 39–65 (50.35)	EG1: 15/18 EG2: 17/17 CG: 17/19	ACR (1995)	EG1: Xian ling gu bao capsule (1.5 g, bid) EG2: EG1 + CG	Sodium hyaluronate (IA, qw, 5 w)	EG1: GS (1) EG2: GS (1) CG: None	VAS-1; VAS-2; WOMAC

Lu et al. (2012) [41]	120/120	EG: 52.93 ± 14.22 CG: 54.01 ± 15.35	EG: 55/65 CG: 50/70	CMA (2007)	Teng huang jian gu pill (1.5–3.0 g, bid, 4 w)	Celecoxib (200 mg, qd, 4 w)	EG: None CG: GS (5)	WOMAC

Li et al. (2012) [42]	58/31	EG: 59.4 ± 10.3 CG: 63.2 ± 14.8	EG: 22/36 CG: 11/20	ACR (1995)	Fufang xiao huo luo pill (2 pills, bid, 2 m)	Glucosamine (250 mg, bid, 2 m)	None	VAS-1; VAS-2; Lysholm

Li et al. (2012) [43]	75/77	EG: 46–73 (55.2) CG: 43–72 (54.1)	EG: 22/53 CG: 23/54	CMA (2007)	Hua mo yan granule (12 g, tid, 4 w) + CG	Infrared therapy (15 min, bid, 4 w)	NR	VAS-1

Jiang et al. (2012) [44]	55/55	EG: 61.92 ± 9.89 CG: 62.78 ± 10.25	EG: 33/22 CG: 31/24	ACR (1986)	Bai shao zong gan capsule (0.3 g, tid, 4 w) + CG	Celecoxib (200 mg, qd, 4 w)	EG: GS (4); ERU (1) CG: GS (2); ERU (1)	VAS-1

Wang et al. (2011) [45]	49/49	56.9 ± 13.0	48/50	ACR (1995)	Jin tian ge capsule (3 pills, tid, 3 m) + CG	Alfacalcidol (0.5 *μ*g, qd) + Naproxen (0.25, bid)	None	Lequesne

Kang et al. (2011) [46]	EG1/EG2/CG: 72/46/42	EG1: 54.34 ± 6.93 EG2: 52.63 ± 7.29 CG: 54.24 ± 6.80	EG1: 14/58 EG2: 10/32 CG: 12/34	ACR (1995)	EG1: Wang bi pill (2.0 g, tid, 8 w) EG2: EG1 + CG	Diclofenac sodium (25 mg, qd/bid, 8 w)	EG1: GS (2) EG2: GS (4) CG: GS (6)	VAS-1

Yan (2010) [47]	30/30	EG: 57.90 ± 7.10 CG: 59.87 ± 7.24	EG: 6/23 CG: 6/24	CMA (2007)	Kang gu zeng sheng capsule (5 pills, tid, 4 w)	Glucosamine (2 pills, tid, 4 w)	EG: None CG: GS (1)	WOMAC

Xiao et al. (2009) [48]	77/77	EG: 56.20 ± 10.64 CG: 57.49 ± 12.82	EG: 21/53 CG: 18/52	NR	Pan long qi pill (3 pills, tid, 6 w)	Diclofenac sodium (25 mg, tid, 6 w)	EG: GS (3); DIZ (2) CG: GS (3)	VAS-2

Qi and Xu (2009) [49]	60/60	EG: 45–69 CG: 46–70	EG: 13/47 CG: 11/49	GCRNDTCM	Xin huang pill (1.28 g, tid, 15 d)	Ibuprofen (0.6 g, bid, 15 d) + Indometacin (25 mg, tid, 15 d)	EG: GS (2) CG: GS (12)	Lysholm

Li (2009) [50]	50/50	EG: 45–68 (51.8) CG: 46–67 (52.6)	EG: 22/28 CG: 23/27	ACR	Bi qi capsule (1.2 g, tid, 6 w)	Glucosamine (250 mg, tid, 6 w)	NR	HSS

H. B. Liu and J. Liu (2007) [51]	66/64	EG: 59.5 ± 9.4 CG: 58 ± 8.5	EG: 23/43 CG: 26/38	ACR	Jin gu tong xiao pill (6 g, bid, 2 m) + CG	Sodium hyaluronate (IA, 5 mL, q4w, 4 w)	None	Lysholm

Chen et al. (2007) [52]	37/34	47–76	24/47	ACR (1986)	Gu jin pill (0.9 g, tid, 8 w)	Diclofenac sodium (75 mg, qd, 8 w)	None	Lysholm

Yang et al. (2006) [53]	60/60	EG: 45–68 (51.2) CG: 46–65 (53.1)	EG: 28/32 CG: 26/34	ACR	Pan long qi pill (3 pills, tid, 6 w)	Glucosamine (250 mg, tid, 6 w)	NR	VAS-1; VAS-2

Xu et al. (2004) [54]	30/30	50–79	25/35	ACR	Bai shao zong gan capsule (0.6 g, bid, 6 w)	Nabumetone (1.0 g, qd, 6 w)	EG: None CG: GS (8)	VAS-1

Note: EG: experimental group; CG: control group; NR: not reported; IA: intra-articular injection; ACR: American College of Rheumatology; CMA: Chinese Medical Association; GCRNDTCM: Guidelines for Clinical Research of New Drugs of Traditional Chinese Medicine; VAS-1: Visual Analog Score-1 (global pain); VAS-2: Visual Analog Score-2 (pain on walking); GS: gastrointestinal symptoms; ALF: abnormal liver function; DM: dry mouth; LS: local swelling; DIZ: dizziness; ERU: eruption.

**Table 2:** Ingredients of manufactured Chinese herbal formulae included in the current review.

MCHF	Components (Latin name [local name])	Registration number
Bai shao zong gan capsule	Extract from Radix paeoniae alba [Bai shao]	National Drug Approval Number H20055058

Bi qi capsule	Semen strychni [Ma qian zi], Pheretima [Di long], Radix codonopsis [Dang shen], Poria [Fu ling], Rhizoma atractylodis macrocephalae [Bai zhu], Radix et rhizoma glycyrrhizae [Gan cao], Rhizoma chuanxiong [Chuan xiong], Radix et rhizoma salvia miltiorrhizae [Dan shen], Radix et rhizoma notoginseng [San qi], and Radix cyathulae [Chuan niu xi]	National Drug Approval Number Z10910026

Fu gui gu tong granule	Radix aconite lateralis praeparata [Fu zi], Radix aconite cocta [Zhi chuan wu], Cortex cinnamomi [Rou gui], Radix codonopsis [Dang shen], Radix angelicae sinensis [Dang gui], Radix paeoniae alba [Bai shao], Folium epimedii [Yin yang huo], and Olibanum [Ru xiang]	National Drug Approval Number Z20000020

Fufang xiao huo luo pill	Radix aconite [Chuan wu], Radix aconite kusnezoffii [Cao wu], Radix angelicae sinensis [Dang gui], Rhizoma chuanxiong [Chuan xiong], Radix paeoniae alba [Bai shao], Pheretima [Di long], Olibanum [Ru xiang], Myrrha [Mo yao], Rhizoma cyperi [Xiang fu], and Bile arisaema cum [Dan nan xing]	National Drug Approval Number Z11020012

Fufang Xiatianwu pill	Rhizoma corydalis decumbentis [Xia tian wu], Radix aconite kusnezoffii [Cao wu], Herba siegesbeckiae [Xi xian cao], Caulis spatholobi [Ji xue teng], Radix et rhizoma clematidis [Wei ling xian], Radix stephaniae tetrandrae [Fang ji], Cortex acanthopanacis [Wu jia pi], Radix et rhizoma notopterygii [Qiang huo], Radix angelicae pubescentis [Du huo], Radix gentianae macrophyllae [Qin jiao], Agkistrodon [Qi she], Herba ephedrae [Ma huang], Radix saposhnikoviae [Fang feng], Scorpio [Quan xie], Batryticatus bombyx [Jiang can], Semen strychni [Ma qian zi], Rhizoma atractylodis [Cang zhu], Olibanum [Ru xiang], Myrrha [Mo yao], Radix aucklandiae [Mu xiang], Rhizoma chuanxiong [Chuan xiong], Radix et rhizoma salvia miltiorrhizae [Dan shen], Radix angelicae sinensis [Dang gui], Radix et rhizoma notoginseng [San qi], Rhizoma drynariae [Gu sui bu], Radix paeoniae rubra [Chi shao], Moschus [She xiang], Syntheticum borneolum [Bing pian], Radix achyranthis bidentatae [Niu xi], and so forth	National Drug Approval Number Z20003105

Gu jin pill	Olibanum [Ru xiang], Myrrha [Mo yao], Radix paeoniae alba [Bai shao], Rhizoma corydalis [Yan hu suo], Radix et rhizoma notoginseng [San qi], Radix aucklandiae [Mu xiang], Flos carthami [Hong hua], Radix curcumae [Yu jin], Radix angelicae pubescentis [Du huo], Radix achyranthis bidentatae [Niu xi], Radix gentianae macrophyllae [Qin jiao], Ramulus cinnamomi [Gui zhi], Sanguis draconis [Xue jie], Semen strychni [Ma qian zi]	National Drug Approval Number Z20003160

Hua mo yan granule	Spica prunellae [Xia ku cao], Fructus ligustri lucidi [Nu zhen zi], Radix astragali [Huang qi], Radix stephaniae tetrandrae [Fang ji], Semen coicis [Yi yi ren], Rhizoma smilacis glabrae [Tu fu ling], Fructus retinervus luffae [Si gua luo], Herba lycopi [Ze lan], Radix et rhizoma salvia miltiorrhizae [Dan shen], Radix angelicae sinensis [Dang gui], Radix achyranthis bidentatae [Niu xi], Herba siegesbeckiae [Xi xian cao], and so forth	National Drug Approval Number Z13020929

Huo xue zhi tong capsule	Radix angelicae sinensis [Dang gui], Radix et rhizoma notoginseng [San qi], Olibanum [Ru xiang], Steleophaga eupolyphaga [Tu bie chong], Pyritum [Zi ran tong], and Syntheticum borneolum [Bing pian]	National Drug Approval Number Z20080118

Jiangu granule	Cortex eucommiae [Du zhong], Radix paeoniae alba [Bai shao], Radix dipsaci [Xu duan], Radix astragali [Huang qi], Fructus lycii [Gou qi zi], Radix achyranthis bidentatae [Niu xi], Radix et rhizoma notoginseng [San qi], Caulis spatholobi [Ji xue teng], Radix et rhizoma ginseng [Ren shen], Radix angelicae sinensis [Dang gui], Cortex phellodendri chinensis [Huang bai], and Radix et rhizoma clematidis [Wei ling xian]	National Drug Approval Number Z20030125

Jin gu tong xiao pill	Radix et rhizoma salvia miltiorrhizae [Dan shen], Caulis spatholobi [Ji xue teng], Rhizoma cyperi [Xiang fu], Radix linderae [Wu yao], Radix cyathulae [Chuan niu xi], Radix gentianae macrophyllae [Qin jiao], Ramulus cinnamomi [Gui zhi], Radix et rhizoma clematidis [Wei ling xian], Radix paeoniae alba [Bai shao], Radix rehmanniae [Di huang], and Radix et rhizoma glycyrrhizae [Gan cao]	National Drug Approval Number Z10970117

Jin tian ge capsule	Powder of artificial tiger bone [Rengong hugu fen]	National Drug Approval Number Z20030080

Kang gu zeng sheng capsule	Radix rehmanniae praeparata [Shu di huang], Caulis spatholobi [Ji xue teng], Herba cistanches [Rou cong rong], Semen raphani [Lai fu zi], Rhizoma cibotii [Gou ji], Rhizoma drynariae [Gu sui bu], Fructus ligustri lucidi [Nv zhen zi], Radix achyranthis bidentatae [Niu xi], and Folium epimedii [Yin yang huo]	National Drug Approval Number Z10980006

Pan long qi pill	Cortex acanthopanacis [Wu jia pi], Cortex eucommiae [Du zhong], Radix angelicae sinensis [Dang gui], Radix gentianae macrophyllae [Qin jiao], Radix aucklandiae [Mu xiang], Caulis et folium trachelospermi [Luo shi teng], Radix aconite [Chuan wu], Radix aconite kusnezoffii [Cao wu], Flos carthami [Hong hua], Myrrha [Mo yao], Herba lycopodii [Shen jin cao], Radix achyranthis bidentatae [Niu xi], Radix et rhizoma salvia miltiorrhizae [Dan shen], Rhizoma paridis [Chong lou], Olibanum [Ru xiang], and so forth	National Drug Approval Number Z61020050

Teng huang jian gu pill	Radix rehmanniae praeparata [Shu di huang], Herba pyrolae [Lu xian cao], Rhizoma drynariae [Gu sui bu], Herba cistanches [Rou cong rong], Folium epimedii [Yin yang huo], Caulis spatholobi [Ji xue teng], and Semen raphani [Lai fu zi]	National Drug Approval Number Z20090570

Wang bi pill	Radix rehmanniae [Di huang], Radix rehmanniae praeparata [Shu di huang], Radix dipsaci [Xu duan], Radix aconite lateralis praeparata [Fu zi], Radix angelicae pubescentis [Du huo], Rhizoma drynariae [Gu sui bu], Ramulus cinnamomi [Gui zhi], Folium epimedii [Yin yang huo], Radix saposhnikoviae [Fang feng], Radix et rhizoma clematidis [Wei ling xian], Spina gleditsiae [Zao jiao ci], Radix paeoniae alba [Bai shao], Rhizoma cibotii [Gou ji], Rhizoma anemarrhenae [Zhi mu], Herba lycopodii [Shen jin cao], and Flos carthami [Hong hua]	National Drug Approval Number Z20044065

Xian ling gu bao capsule	Folium epimedii [Yin yang huo], Radix dipsaci [Xu duan], Fructus psoraleae [Bu gu zhi], Radix rehmanniae [Di huang], Radix et rhizoma salvia miltiorrhizae [Dan shen], and Rhizoma anemarrhenae [Zhi mu]	National Drug Approval Number Z20025337

Xin huang pill	Herba sarcandrae [Zhong jie feng], Radix et rhizoma notoginseng [San qi], Calculus bovis artifactus [Rengong niuhuang], and so forth	National Drug Approval Number Z35020063

*Note*. Registration number was acquired from the administration's website of CFDA (http://eng.sfda.gov.cn/WS03/CL0755/).

**Table 3:** Methodological quality of the included studies.

Study ID	Random sequence generation	Allocation concealment	Blinding of participants and personnel	Blinding of outcome assessment	Incomplete outcome data	Selective reporting	Other bias
Zuo et al. (2015) [29]	Low risk	Low risk	High risk	High risk	Low risk	Unclear risk	Unclear risk
Huang and Quan (2015) [30]	Unclear risk	Unclear risk	Low risk	Unclear risk	Low risk	Unclear risk	Unclear risk
Cao et al. (2015) [31]	Low risk	Unclear risk	Unclear risk	Unclear risk	Low risk	Unclear risk	Unclear risk
Zhang et al. (2013) [32]	Unclear risk	Unclear risk	Unclear risk	Unclear risk	Low risk	Unclear risk	Unclear risk
Yi and Ji (2013) [33]	Unclear risk	Unclear risk	Unclear risk	Unclear risk	Low risk	Unclear risk	Unclear risk
Niu et al. (2013) [34]	Low risk	Unclear risk	Unclear risk	Unclear risk	Low risk	Unclear risk	Unclear risk
Liao (2013) [35]	Low risk	Unclear risk	Unclear risk	Unclear risk	Low risk	Unclear risk	Unclear risk
Li et al. (2013) [36]	Unclear risk	Unclear risk	Unclear risk	Unclear risk	Low risk	Unclear risk	Unclear risk
Li et al. (2013) [37]	Unclear risk	Unclear risk	Unclear risk	Unclear risk	Low risk	Unclear risk	Unclear risk
Li et al. (2013) [38]	Low risk	Unclear risk	Unclear risk	Unclear risk	Low risk	Unclear risk	Unclear risk
Chen et al. (2013) [39]	Low risk	Unclear risk	Unclear risk	Unclear risk	Low risk	Unclear risk	Unclear risk
Luo et al. (2012) [40]	Low risk	Unclear risk	Unclear risk	Unclear risk	Low risk	Unclear risk	Unclear risk
Lu et al. (2012) [41]	Low risk	Unclear risk	Unclear risk	Unclear risk	Low risk	Unclear risk	Unclear risk
Li et al. (2012) [42]	Unclear risk	Unclear risk	Unclear risk	Unclear risk	Low risk	Unclear risk	Unclear risk
Li et al. (2012) [43]	Unclear risk	Unclear risk	Unclear risk	Unclear risk	Low risk	Unclear risk	Unclear risk
Jiang et al. (2012) [44]	Low risk	Unclear risk	Unclear risk	Unclear risk	Low risk	Unclear risk	Unclear risk
Wang et al. (2011) [45]	Low risk	Unclear risk	Unclear risk	Unclear risk	Low risk	Unclear risk	Unclear risk
Kang et al. (2011) [46]	Low risk	Unclear risk	Unclear risk	Unclear risk	Low risk	Unclear risk	Unclear risk
Yan (2010) [47]	Unclear risk	Unclear risk	Unclear risk	Unclear risk	Low risk	Unclear risk	Unclear risk
Xiao et al. (2009) [48]	Low risk	Unclear risk	Unclear risk	Unclear risk	Low risk	Unclear risk	Unclear risk
Qi and Xu (2009) [49]	Low risk	Unclear risk	Unclear risk	Unclear risk	Low risk	Unclear risk	Unclear risk
Li (2009) [50]	Unclear risk	Unclear risk	Unclear risk	Unclear risk	Low risk	Unclear risk	Unclear risk
H. B. Liu and J. Liu (2007) [51]	Unclear risk	Unclear risk	Unclear risk	Unclear risk	Low risk	Unclear risk	Unclear risk
Chen et al. (2007) [52]	High risk	Unclear risk	Unclear risk	Unclear risk	Low risk	Unclear risk	Unclear risk
Yang et al. (2006) [53]	Unclear risk	Unclear risk	Unclear risk	Unclear risk	Low risk	Unclear risk	Unclear risk
Xu et al. (2004) [54]	Unclear risk	Unclear risk	Unclear risk	Unclear risk	Low risk	Unclear risk	Unclear risk

## References

[1] Felson D. T., Lawrence R. C., Dieppe P. A. (2000). Osteoarthritis: new insights. Part 1: the disease and its risk factors. *Annals of Internal Medicine*.

[2] Dieppe P. A., Lohmander L. S. (2005). Pathogenesis and management of pain in osteoarthritis. *The Lancet*.

[3] Zhang Y., Jordan J. M. (2010). Epidemiology of osteoarthritis. *Clinics in Geriatric Medicine*.

[4] Lawrence R. C., Felson D. T., Helmick C. G. (2008). Estimates of the prevalence of arthritis and other rheumatic conditions in the United States. Part II. *Arthritis and Rheumatism*.

[5] Barten D. J., Swinkels l. C., Dorsman S. A., Dekker J., Veenhof C., de Bakker D. H. (2015). Treatment of hip/knee osteoarthritis in Dutch general practice and physical therapy practice: an observational study. *BMC Family Practice*.

[6] Silverwood V., Blagojevic-Bucknall M., Jinks C., Jordan J. L., Protheroe J., Jordan K. P. (2015). Current evidence on risk factors for knee osteoarthritis in older adults: a systematic review and meta-analysis. *Osteoarthritis and Cartilage*.

[7] Woolf A. D., Pfleger B. (2003). Burden of major musculoskeletal conditions. *Bulletin of the World Health Organization*.

[8] Hochberg M. C., Altman R. D., April K. T. (2012). American College of Rheumatology 2012 recommendations for the use of nonpharmacologic and pharmacologic therapies in osteoarthritis of the hand, hip, and knee. *Arthritis Care and Research*.

[9] Fernandes L., Hagen K. B., Bijlsma J. W. J. (2013). EULAR recommendations for the non-pharmacological core management of hip and knee osteoarthritis. *Annals of the Rheumatic Diseases*.

[10] Brown G. A. (2013). AAOS clinical practice guideline: treatment of osteoarthritis of the knee: evidence-based guideline, 2nd edition. *Journal of the American Academy of Orthopaedic Surgeons*.

[11] Bannuru R. R., Schmid C. H., Kent D. M., Vaysbrot E. E., Wong J. B., McAlindon T. E. (2015). Comparative effectiveness of pharmacologic interventions for knee osteoarthritis: a systematic review and network meta-analysis. *Annals of Internal Medicine*.

[12] Zeng C., Wei J., Li H. (2015). Comparison between 200 mg QD and 100 mg BID oral celecoxib in the treatment of knee or hip osteoarthritis. *Scientific Reports*.

[13] Essex M. N., O'Connell M. A., Behar R., Bao W. (2015). Efficacy and safety of nonsteroidal anti-inflammatory drugs in Asian patients with knee osteoarthritis: summary of a randomized, placebo-controlled study. *International Journal of Rheumatic Diseases*.

[14] Kim T.-H., Kim K. H., Kang J. W. (2014). Moxibustion treatment for knee osteoarthritis: a multi-centre, non-blinded, randomised controlled trial on the effectiveness and safety of the moxibustion treatment versus usual care in knee osteoarthritis patients. *PLoS ONE*.

[15] Shen L. L., Huang G. F., Tian W. (2015). Electroacupuncture inhibits chronification of the acute pain of knee osteoarthritis: study protocol for a randomized controlled trial. *Trials*.

[16] Cortés Godoy V., Gallego Izquierdo T., Lázaro Navas I., Pecos Martín D. (2014). Effectiveness of massage therapy as co-adjuvant treatment to exercise in osteoarthritis of the knee: a randomized control trial. *Journal of Back and Musculoskeletal Rehabilitation*.

[17] Lao L., Hochberg M., Lee D. (2015). Huo-Luo-Xiao-Ling (HLXL)-Dan, a Traditional Chinese Medicine, for patients with osteoarthritis of the knee: a multi-site, randomized, double-blind, placebo-controlled phase II clinical trial. *Osteoarthritis and Cartilage*.

[18] Chen R., Chen M., Su T. (2015). Heat-sensitive moxibustion in patients with osteoarthritis of the knee: a three-armed multicentre randomised active control trial. *Acupuncture in Medicine*.

[19] Ren X., Yao C., Wu F., Li Z., Xing J., Zhang H. (2015). Effectiveness of moxibustion treatment in quality of life in patients with knee osteoarthritis: a randomized, double-blinded, placebo-controlled trial. *Evidence-Based Complementary and Alternative Medicine*.

[20] Yao S., Zhang J., Wang D. (2015). Discriminatory components retracing strategy for monitoring the preparation procedure of Chinese patent medicines by fingerprint and chemometric analysis. *PLoS ONE*.

[21] Yang P., He X., Yang Z., Liu D., Li H., Wang D. (2004). Effects of bushen huoxue decoction on nitric oxide (NO) in serum, articular cartilage and synovium in rabbits of knee osteoarthritis. *Journal of Traditional Chinese Medicine*.

[22] Chen C.-W., Sun J., Li Y.-M., Shen P.-A., Chen Y.-Q. (2011). Action mechanisms of Du-Huo-Ji-Sheng-Tang on cartilage degradation in a rabbit model of osteoarthritis. *Evidence-Based Complementary and Alternative Medicine*.

[23] Cameron M., Chrubasik S. (2014). Oral herbal therapies for treating osteoarthritis. *Cochrane Database of Systematic Reviews*.

[24] Wang X., Wei S., Liu T. (2014). Effectiveness, medication patterns, and adverse events of traditional Chinese herbal patches for osteoarthritis: a systematic review. *Evidence-Based Complementary and Alternative Medicine*.

[25] Hochberg M. C., Altman R. D., Brandt K. D. (1995). Guidelines for the medical management of osteoarthritis. Part II. Osteoarthritis of the knee. *Arthritis and Rheumatism*.

[26] Chinese Medical Association of Orthopaedics (2007). Osteoarthritis guidelines of diagnosis and treatment. *Chinese Journal of Joint Surgery*.

[27] Zhang W., Doherty M., Peat G. (2010). EULAR evidence-based recommendations for the diagnosis of knee osteoarthritis. *Annals of the Rheumatic Diseases*.

[28] McAlindon T. E., Bannuru R. R., Sullivan M. C. (2014). OARSI guidelines for the non-surgical management of knee osteoarthritis. *Osteoarthritis and Cartilage*.

[29] Zuo C., Yin G., Cen X. M., Xie Q. B. (2015). Controlled clinical study on compound Decumbent corydalis rhizome and diclofenac in treatment of knee osteoarthritis. *China Journal of Chinese Materia Medica*.

[30] Huang F., Quan Y. H. (2015). Clinical observation of Huo xue zhi tong soft capsule combined with sodium hyaluronate in treatment of knee osteoarthritis. *Journal of Hubei University of Chinese Medicine*.

[31] Cao J. G., Wang T. Y., Wang L. (2015). The clinical research of Chinese traditional medicine Jin tian ge capsule for the treatment of osteoarthritis. *Chinese Journal of Osteoporosis*.

[32] Zhang J. L., Ren B. D., Wang Y. Clinical observation on 63 cases of knee osteoarthritis treating with Pan Long Qi pill.

[33] Yi L., Ji H. W. Clinical observation of Pan Long Qi pill in treatment of knee osteoarthritis.

[34] Niu L., Zhang J. S., Liang C. M. (2013). Clinical study on 27 cases of knee osteoarthritis treating with Jin Gu Tong Xiao pill plus glucosamine sulfate. *Jiangxi Journal of Traditional Chinese Medicine*.

[35] Liao W. Q. (2013). Clinical observation of knee osteoarthritis treating with Jiangu granulate plus sodium hyaluronate. *Journal of New Chinese Medicine*.

[36] Li P. F., Jin X. H., Zhang Q. S. (2013). Clinical observation on Hua mo yan granule for the treatment of vocational knee osteoarthritis. *China Health Care and Nutrition*.

[37] Li P. F., Jin X. H., Zhang Q. S., Cui S. J., Yu T. M., Wei W. (2013). Clinical observation on Hua mo yan granule plus infrared for the treatment of knee osteoarthritis. *Guide of China Medicine*.

[38] Li J.-H., Zhou L.-X., Li G.-Y., Cheng B. (2013). Treatment of middle-aged and aged patients with knee osteoarthritis of yang-deficiency induced cold-damp syndrome by ozone combined Chinese materia medica: a clinical research. *Chinese Journal of Integrated Traditional and Western Medicine*.

[39] Chen S. C., Hu Y. X., Lai J. C. (2013). Clinical effect of medical ozone combined with Xian Ling Gu Bao for knee osteoarthritis. *Hainan Medical Journal*.

[40] Luo Y., Guo M. Y., Zhang J. (2012). Clinical observation on Xian Ling Gu Bao plus sodium hyaluronate for the treatment of knee osteoarthritis. *Modern Journal of Integrated Traditional Chinese and Western Medicine*.

[41] Lu M., Zhang B., Zou Z., Xu W. J. (2012). Multi-center clinical observation of Teng huang jian gu tablets in the treatment to osteoarthritis of the knee typed with diffident kidney and blood stasis. *Chinese Journal of Traditional Medical Traumatology and Orthopeics*.

[42] Li T. H., Luo S. F., Yang S. Y. (2012). Clinical study on 58 cases of knee osteoarthritis treating with Xiao Huo Luo pill. *Henan Traditional Chinese Medicine*.

[43] Li P. F., Jin X. H., Zhang Q. S., Cui S. J. (2012). Clinical observation on Huamoyan granule and infrared ray for the treatment of knee osteoarthritis. *Guide of China Medicine*.

[44] Jiang Q., Jin X. F., Zhu P. J. (2012). Clinical observation on celecoxib plus Bai Shao Zong Gan capsule for the treatment of knee osteoarthritis. *Strait Pharmaceutical Journal*.

[45] Wang J. P., Zhang J. H., Wang H. J., Yang D., Zhang S. G. (2011). Analysis on the clinical efficacy of Jin tian ge capsule for the treatment of osteoarthritis. *Chinese Journal of Hospital Pharmacy*.

[46] Kang X.-Z., Wu Q.-F., Jie H.-Y. (2011). Clinical study on the treatment of knee osteoarthritis by Wang bi tablet. *Chinese Journal of Integrated Traditional and Western Medicine*.

[47] Yan X. G. *Clinical study on treatment of mild-to-moderate knee osteoarthritis by anti-hyperosteogeny capsule and Shishi manipulation [M.S. thesis]*.

[48] Xiao L. J., Deng D. L., Chen W. G., Xu H. (2009). Therapeutic effect of Pan Long Qi pill on osteoarthritis of knee joint. *China Foreign Medical*.

[49] Qi K. Z., Xu W. J. (2009). Clinical study on the treatment of knee osteoarthritis by Xin Huang pill. *Chinese Journal of Traditional Medical Rheumatology*.

[50] Li L. (2009). Clinical study on the treatment of knee osteoarthritis by Bi Qi capsule. *China Journal of Traditional Chinese Medicine and Pharmacy*.

[51] Liu H. B., Liu J. (2007). Clinical study on the treatment of knee osteoarthritis by sodium hyaluronate plus Jin Gu Tong Xiao pill. *The Journal of Traditional Chinese Orthopedics and Traumatology*.

[52] Chen P. B., Jia H., Deng Y. J. (2007). Clinical study on the treatment of knee osteoarthritis by Gu Jin pill. *Xinjiang Journal of Traditional Chinese Medicine*.

[53] Yang Z. P., Li X., Han J., Li Z. F. (2006). Therapeutic effect of Pan Long Qi pill on osteoarthritis of knee joint and comparative study. *Chinese Journal of Traditional Medical Traumatology and Orthopeics*.

[54] Xu F. Y., He C. S., Gan J. H., Yang D. J., Nie W. S. (2004). Curative effectiveness and safety of total glucosides of Paeony in the treatment of knee osteoarthritis. *Chinese Journal of Rehabilitation*.

[55] Liberati A., Altman D. G., Tetzlaff J. (2009). The PRISMA statement for reporting systematic reviews and meta-analyses of studies that evaluate healthcare interventions: explanation and elaboration. *British Medical Journal*.

[56] Streiner D., Geddes J. (2001). Intention to treat analysis in clinical trials when there are missing data. *Evidence-Based Mental Health*.

[57] Little R., Kang S. (2015). Intention-to-treat analysis with treatment discontinuation and missing data in clinical trials. *Statistics in Medicine*.

[58] Higgins J. P. T., Green S. (2011). *Cochrane Handbook for Systematic Reviews of Interventions, Version 5.1.0*.

[59] Higgins J. P. T., Green S. Cochrane Reviewers' Handbook 5.1.0. http://handbook.cochrane.org/.

[60] DerSimonian R., Laird N. (1986). Meta-analysis in clinical trials. *Controlled Clinical Trials*.

[61] Higgins J. P. T., Thompson S. G. (2002). Quantifying heterogeneity in a meta-analysis. *Statistics in Medicine*.

[62] Veale D., Miles S., Smallcombe N., Ghezai H., Goldacre B., Hodsoll J. (2014). Atypical antipsychotic augmentation in SSRI treatment refractory obsessive-compulsive disorder: a systematic review and meta-analysis. *BMC Psychiatry*.

[63] Sterne J. A. C., Gavaghan D., Egger M. (2000). Publication and related bias in meta-analysis: power of statistical tests and prevalence in the literature. *Journal of Clinical Epidemiology*.

[64] Zhu L., Gao J., Yu J. (2015). Jingtong granule: a chinese patent medicine for cervical radiculopathy. *Evidence-Based Complementary and Alternative Medicine*.

[65] Zhou L., Guo S. N., Gao Y. (2015). Effects and perspectives of Chinese patent medicines for tonifying Qi and promoting blood circulation on patients with cerebral infarction. *Current Vascular Pharmacology*.

[66] Zhao Y., Du B., Jiang X. (2014). Effects of combining lowdose aspirin with a Chinese patent medicine on follicular blood flow and pregnancy outcome. *Molecular Medicine Reports*.

[67] Xiong X., Wang P., Zhang Y., Li X. (2015). Effects of traditional Chinese patent medicine on essential hypertension: a systematic review. *Medicine*.

[68] Lei X., Chen J., Liu C.-X., Lin J., Lou J., Shang H.-C. (2014). Status and thoughts of Chinese patent medicines seeking approval in the US market. *Chinese Journal of Integrative Medicine*.

[69] Wang X., Wang Z.-F., Xie Y.-M., Zhang W., Liao X., Chang Y.-P. (2015). Guideline for postmarketing Chinese medicine pharmacoeconomic evaluation. *Chinese Journal of Integrative Medicine*.

[70] Zhang L.-G., Ouyang X.-W., Wu T.-T., Ni L.-J., Shi W.-Z. (2014). Quantitative evaluation of *in vitro* effects and interactions of active fractions in a Chinese medicinal formula (Yaotongning Capsule) on rat chondrocytes. *Journal of Ethnopharmacology*.

[71] Li X., Liu F., Liang W. (2014). Tougu Xiaotong capsule promotes chondrocyte autophagy by regulating the Atg12/LC3 conjugation systems. *International Journal of Molecular Medicine*.

[72] Wang X., Cao Y., Pang J. (2012). Traditional Chinese herbal patch for short-term management of knee osteoarthritis: a randomized, double-blind, placebo-controlled trial. *Evidence-Based Complementary and Alternative Medicine*.

[73] Wang F. D., Guo Y. J., Dong L. X., Wang Z. (2012). Effects of Kanggu Zengsheng tablets on bFGF protein and mRNA expression in rabbit chondrocytes with knee osteoarthritis. *Chinese Journal of Traditional Medical Traumatology and Orthopedics*.

[74] Li F., Yao J. H., Zhang F. X. (2014). Effects of Biqi Capsule on the expression of TIMP-1 and MMP-3 in patients with knee osteoarthritis. *China Journal of Traditional Chinese Medicine and Pharmacy*.

